# Urinary phenols and parabens exposure in relation to urinary incontinence in the US population

**DOI:** 10.1186/s12889-024-17872-9

**Published:** 2024-02-19

**Authors:** Jinjiang Jiang, Bo Chen, Bo Tang, Jinze Li, Chensong Zhang, Daqing Tan, Ting Zhang, Qiang Wei

**Affiliations:** 1 Department of Urology, Institute of Urology, West China Hospital of Sichuan University, No. 37, Guoxue Lane, Chengdu, Sichuan China; 2 State Key Laboratory of Biotherapy and Cancer Center, Frontiers Science Center for Disease-Related Molecular Network, and National Clinical Research Center for Geriatrics, Sichuan University, Chengdu, Sichuan China; 3 School of Basic Medicine, Harbin Medical Hospital, Harbin, China

**Keywords:** Urinary incontinence, Personal care products, triclosan, bisphenol A, benzophenone-3, Parabens

## Abstract

**Background:**

Our study aimed to investigate the impact of urinary concentrations of personal care products (PCPs)-related phenols (PNs) and parabens (PBs), including Triclosan (TCS), Bisphenol A (BPA), Benzophenone-3 (BP-3), Butylparaben (BPB), Ethylparaben (EPB), Methylparaben (MPB), and Propylparaben (PPB), on urinary incontinence (UI) occurrence.

**Method:**

We conducted a cross-sectional analysis using data from the National Health and Nutrition Examination Survey (NHANES) spanning the years 2007 to 2016. Regression analysis was employed to investigate the relationship between exposure to PCPs-related substances, various levels of exposure, and UI within both the general population and the female demographic. Additionally, the Bayesian Kernel Machine Regression (BKMR) model was used to assess the effects of mixtures on UI.

**Results:**

Our analysis comprised 7,690 participants who self-reported their diagnosis. Among them, 12.80% experienced stress urinary incontinence (SUI), 11.80% reported urge urinary incontinence (UUI), and 10.22% exhibited mixed urinary incontinence (MUI). In our fully adjusted multivariable models, BP-3 exposure exhibited a positive association with SUI (OR 1.07, 95% CI 1.02–1.14, *p* = 0.045). BPA exposure correlated with an increased risk of UUI (OR 1.21, 95% CI 1.01–1.44, *p *= 0.046) and MUI (OR 1.26, 95% CI 1.02–1.54, *p* = 0.029). TCS exposure displayed a negative correlation with the incidence of MUI (OR 0.87, 95% CI 0.79–0.97, *p* = 0.009). No significant links were observed between parabens and urinary incontinence. Notably, among the female population, our investigation revealed that BPA exposure heightened the risk of MUI (OR 1.28, 95% CI 1.01–1.63, *p* = 0.043). Participants in the highest tertile of BP-3 exposure demonstrated elevated likelihoods of SUI and MUI compared to those in the lowest tertile. In the BKMR analysis, negative trends were observed between the mixture and the risks of UUI and MUI when the mixture ranged from the 25th to the 40th and 35th to the 40th percentiles or above, respectively. Additionally, a positive trend was identified between the mixture and MUI when it was in the 40th to 55th percentile.

**Conclusion:**

In conclusion, our findings suggest that exposure to BPA, TCS, and BP-3 may contribute to the development of urinary incontinence.

**Supplementary Information:**

The online version contains supplementary material available at 10.1186/s12889-024-17872-9.

## Introduction

Urinary incontinence (UI) is characterized by the involuntary loss of urine, resulting in an individual's inability to control the timing and amount of urine expelled. According to Irwin et al., approximately 13.1% of women and 5.4% of men have experienced UI [1]. Furthermore, persistent UI significantly impacts the quality of life. Gibson's research reveals that elderly women with incontinence have a 1.5 to 2.3-fold higher risk of falls, contributing to increased overall morbidity, mortality, and healthcare expenditure [2]. These findings highlight UI as a prevalent issue with profound implications for health-related quality of life in the general population.

The intricate nature of UI emphasizes the pivotal role of pelvic floor integrity in its pathogenesis, which involves factors such as detrusor overactivity, poor detrusor compliance, bladder hypersensitivity, and urethral hypermobility [3]. Research indicates that menopausal women with SUI exhibit reduced estrogen receptors in the periurethral fascia. Hormonal shifts among SUI patients alter extracellular matrix components, contributing to SUI by affecting tissue architecture and mechanical attributes [4, 5]. Additionally, ongoing urothelial irritation due to local immuno-inflammatory responses can contribute to urinary incontinence [3]. This implies that changes in the endocrine state and inflammation can impact pelvic floor tissue structure, leading to urinary dysfunction. Besides, environmental factors, such as organophosphate esters with known endocrine-disrupting properties, are also linked to structural disruptions in the pelvic floor, contributing to incontinence [6].

Daily use of PCPs exposes individuals to environmental pollutants, including triclosan (TCS), benzophenone-3 (BP-3), bisphenol A (BPA), and parabens (PBs). Benzophenone-3 is primarily sourced from UV filters in sunscreen formulations and many other consumer products [7]. Furthermore, Pycke et al. [8] suggest that TCS exposure primarily occurs through the topical application of PCPs, dermal contact with consumer products, and toothpaste ingestion, representing a significant exposure route for human populations. PBs, a group of alkyl esters (e.g., methyl, ethyl, propyl, butyl) of hydroxybenzoic acid, are widely employed as antimicrobial preservatives in personal care items, and studies have identified PCPs as significant sources of PBs exposure [9]. BPA is integral to the manufacture of polycarbonate plastics and epoxy resins [10]. While exposure from PCPs may be minimal, we encapsulate the entirety under the term "personal care products related phenols" for a comprehensive analysis of PCPs-related substances.

These compounds, known for their potential bioactive effects, may contribute to diseases by disrupting normal biological processes [3]. Notably, as endocrine disruptors, TCS and parabens hinder human aromatase activity, reducing estrogen production [11]. BPA's strong interaction with estrogen receptors (ER) is recognized to impair ER functionality [12]. An in-depth analysis of BP-3 suggests that its highest internal concentrations, attained after a single application of commercially available sunscreen (4% w/w), align with concentrations causing endocrine-disrupting effects in vitro and adverse effects on female reproduction in rodents in vivo [13]. Beyond endocrine disruption, Aung et al. [14] found inverse relationships between maternal plasma inflammatory markers and urinary PNs and PBs levels, and Watkins et al. [15] identified a significant link between elevated urinary BPA and parabens levels and increased oxidative stress indicators. Long-term exposure to bisphenol A in mice was associated with urothelium impact, resulting in increased prostatic ducts and reduced urethra lumen size [16].

Considering the potential implications of the mentioned PNs and PBs on pathways related to UI, exposure to these environmental pollutants may adversely affect pelvic tissues. Our study is the first to explore the biological impact of PCPs-related substances on pelvic floor diseases. Through demographic data analysis, we aim to illuminate pelvic floor structural disorders and environmental influences, providing valuable clinical evidence and suggesting innovative treatment strategies.

## Materials and Methods

We adhere to the NHANES Survey Methods and Analytic Guidelines for data collection and processing, available at https://wwwn.cdc.gov/nchs/nhanes/analyticguidelines.aspx.

### Study population

**Fig. 1:**
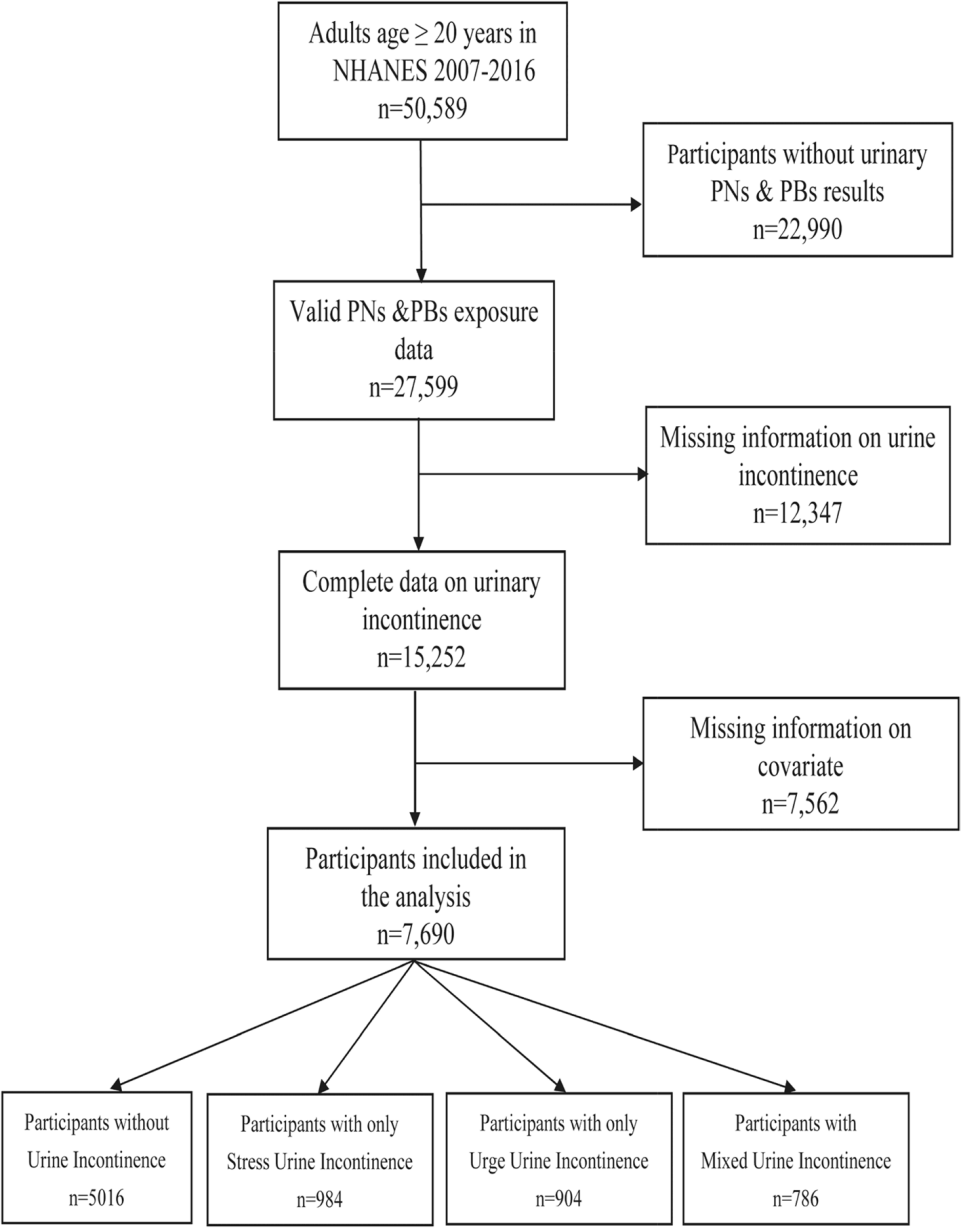
Flow chart of the selection of eligible participants, NHANES 2007–2016

The National Health and Nutrition Examination Survey (NHANES) is an ongoing cross-sectional dataset that represents a sample of the noninstitutionalized U.S. population, offering confidential, voluntary participation to civilians. The study population is nationally representative, recruited through a multistage, stratified sampling design. We utilized publicly available data from participants recruited between 2007 and 2016, specifically including individuals aged 20 years and older with complete demographic information, health profiles, biological samples, and physical evaluations. This resulted in a final analysis cohort of 7690 participants (Fig. 1).

### Parabens and Phenols exposure assessment

These samples were processed by the Division of Laboratory Sciences, Organic Analytical Toxicology Branch at the National Center for Environmental Health (https://www.cdc.gov/nceh/dls/oatb_capacity_13.html). The limits of detection in urine for BPA, BP3, TRS, BUP, EPB, PPB, and MPB are 0.4, 0.4, 2.3, 0.2, 1, 0.2, and 1 ng/ml, respectively. Concentrations below the limit of detection (LOD) were imputed as LOD divided by the square root of two [17]. Furthermore, PN and PB concentrations were adjusted based on urinary creatinine levels (presented as 100 mg/ml creatinine) to account for urine dilution. Due to non-normal distribution, urinary chemical concentrations were log10-transformed. Samples were classified into three groups: undetectable levels (low exposure), detectable levels below the median (median exposure), and detectable levels above the median (high exposure).

### Urinary incontinence assessment

Trained health technicians from NHANES used established criteria to determine the presence of urinary incontinence within the preceding 12 months through two questions. Stress urinary incontinence was queried with, ‘Have you experienced urine leakage during activities such as coughing, lifting, or exercise?’ Urge urinary incontinence was assessed with, ‘Have you experienced urine leakage due to an urgent sensation or pressure to urinate, but were unable to reach a restroom in time?’ Affirmative responses indicated the presence of the respective type of urinary incontinence. Mixed urinary incontinence was diagnosed when both stress and urge urinary incontinence symptoms were present.

### Covariates

Covariates included numerical and categorical variables: gender, age, race, body mass index (BMI), education level, caffeine and water intake, comorbidity index, physical activity, alcohol use, smoking status, history of hysterectomy, estrogen use, and vaginal deliveries. Ethnicity was categorized as Mexican American, Other Hispanic, Non-Hispanic White, Non-Hispanic Black, and other Race. BMI was calculated as weight (kg) divided by height meters squared (m2) and classified into normal weight (< 25), overweight (25 to < 30), and obesity (≥ 30). Education levels encompassed categories of Less Than 9th Grade, 9th-11th Grade, High School Grade, Some College, College Graduate or above. Caffeine and water intake were assessed through two 24-h dietary recall interviews. The comorbidity index quantified disease risk by considering the history of congestive heart failure, asthma, stroke, diabetes, and memory problems, ranging from 0 to 3. A score of 0 indicated no comorbidities, 1 represented one comorbidity, 2 indicated two comorbidities, and 3 denoted three to five comorbidities. Additionally, participants reported their physical activity during a typical week, categorized into less than moderate, moderate, or vigorous based on intensity, using the Global Physical Activity Questionnaire provided by NHANES. Smoking status was classified as never or ever smokers. Participants were categorized as frequent alcohol users or never-drinkers. Vaginal deliveries were classified as 0–3, denoting the number of deliveries. All these covariates were included in the adjusted model, as they have been previously reported as risk factors for urinary incontinence in the general population.

### Statistical analysis

Differences in variables between groups with and without urinary incontinence were assessed using chi-square (categorical) and Kruskal–Wallis H tests (continuous).

We employed weighted multivariate logistic regression models (Crude, Model 1, Model 2, and Model 3) to assess the association between urinary incontinence and PNs and PBs exposure, presenting odds ratios (ORs) and 95% confidence intervals (CIs) for stress, urge, and mixed incontinence. Adjustments in the models varied: Crude model had no adjustments; Model 1 adjusted for age, gender, race, education, BMI; Model 2 adjusted for age, gender, race, education, BMI, comorbidity index, alcohol use, smoking status, caffeine intake, total water intake, and physical activity; Model 3 adjusted for age, race, education, BMI, comorbidity index, alcohol use, smoking status, caffeine intake, total water intake, physical activity, vaginal deliveries, hysterectomy, and hormone use. Trend tests and subgroup analyses stratified by age, BMI, alcohol use, smoking status, and physical activity intensity were conducted. Considering the potential non-linear and non-additive dose–response relationships among mixture exposure; we employed Bayesian kernel machine regression (BKMR) to evaluate the combined effect of PNs and PBs on UI risk. This method is known for its flexibility in exposure–response function modeling and visualizes the impact of the impact of combined exposure [18].

Statistical significance was set at *p* < 0.05, using two-tailed tests. All analyses were performed using R and EmpowerStats. Our weighted analysis reflects USA population cycles.

## Results

### Participant Characteristics

A total of 7,690 participants were included in our study, among whom 984 (12.8%) reported Stress Urinary Incontinence (SUI), 904 (11.8%) reported Urge Urinary Incontinence (UUI), and 786 (10.22%) reported Mixed Urinary Incontinence (MUI) (Table 1). Older non-Hispanic women exhibited a higher prevalence of urinary incontinence. Participants with any type of urinary incontinence tended to possess lower educational attainment, higher Body Mass Index (BMI), and greater comorbidity index compared to those without urinary incontinence. Other factors significantly associated with urinary incontinence included Physical Activity, Alcohol Use, Smoking Status, Vaginal Deliveries, Hormone Use History and Hysterectomy.

**Table 1:** Descriptive statistics for urinary incontinence status and selected covariates among general population ≥ 20 years of age; NHANES 2007–2016

	Stress Urine Incontinence			Urge Urine Incontinence			Mixed Urine Incontinence		
	No	Yes	*P* value	No	Yes	*P* value	No	Yes	*P* value
**Number**	5016	984		5016	904		5016	786	
**Age(years),mean(SD)**	46.0 ± 17.60	50.3 ± 15.7	< 0.001	46.0 ± 17.6	58.4 ± 16.5	< 0.001	46.0 ± 17.6	57.20 ± 15.60	< 0.001
**Gender (n,%)**			< 0.001			< 0.001			< 0.001
Male	3169(63.2%)	68(6.9%)		3169(63.2%)	474(52.4%)		3369(63.2%)	108(13.7%)	
Female	1847(36.8%)	916(93.1%)		1847(36.8%)	430(47.6%)		1847(36.8%)	678(86.3%)	
**Race (n,%)**			< 0.001			< 0.001			0.001
Mexican American	753(15.0%)	179(18.2%)		753(15.0%)	120(13.3%)		753(15.0%)	120(15.3%)	
Other Hispanic	542(10.8%)	113(11.5%)		542(10.8%)	76(8.4%)		542(10.8%)	85(10.8%)	
Non-Hispanic White	2097(41.8%)	470(47.8%)		2097(41.8%)	357(39.5%)		2097(41.8%)	372(47.3%)	
Non-Hispanic Black	1043(20.8%)	143(14.5%)		1043(20.8%)	308(34.1%)		1.43(20.8%)	154(19.6%)	
Other Race	581(11.6%)	79(8.0%)		581(11.6%)	43(4.8%)		581(11.6%)	55(7.00%)	
**BMI (Kg/m**^**2**^**,%)**			< 0.001			< 0.001			< 0.001
normal	1562(31.2%)	260(26.4%)		1564(31.2%)	205(22.7%)		1564(31.2%)	161(20.5%)	
overweight	1750(34.9%)	298(30.3%)		1750(34.9%)	272(30.1%)		1750(34.9%)	210(26.7%)	
obesity	1702(33.9%)	426(43.3%)		1702(33.9%)	427(47.2%)		1702(33.9%)	415(52.80%)	
**Education (n,%)**			0.002			< 0.001			< 0.001
Less Than 9th Grade	457(9.1%)	102(10.4%)		457(9.1%)	101(11.2%)		457(9.1%)	104(13.2%)	
9-11th Grade	727(14.5%)	115(11.7%)		727(14.5%)	144(15.9%)		727(14.5%)	160(20.4%)	
High School Grade	1157(23.1%)	201(20.4%)		1157(23.1%)	230(25.2%)		1157(23.1%)	179(22.8%)	
Some College or AA degree	1407(28.1%)	328(33.3%)		1407(28.1%)	264(29.2%)		1407(28.1%)	217(27.6%)	
College Graduate or above	1268(25.3%)	238(24.2%)		1268(25.3%)	165(18.3%)		1268(25.3%)	126(16.0%)	

The results of age, caffein intake and total water intake are presented as Mean ± SD.

The results of gender, race, BMI, education, comordity index, alcohol use, smoking status, physical activity, vaginal deliveries, hormone use history, hysterectomy are presented as number(percent%).

### PNs and PBs levels in urine samples

Table 2 outlines the means and statistical characteristics of PNs and PBs categorized by urinary incontinence status. Substantial urinary concentration disparities in PNs and PBs were observed between incontinence patients and non-patients. Specifically, mean levels of BPA, Methylparaben, and Propylparaben were significantly elevated among UUI patients. Mean PNs and PBs levels, except for TCS, were notably higher among the MUI population compared to noncases.

**Table 2:** Differences of urinary phenols and paraben concentrations (100 mg/g creatinine) among general population ≥ 20 years of age; NHANES 2007–2016

	Stress Urine Incontinence			Urge Urine Incontinence			Mixed Urine Incontinence		
	No	Yes	*P* value	No	Yes	*P* value	No	Yes	*P* value
Number	5016	984		5016	904		5016	786	
Benzophenone-3	3.65 ± 1.14	3.94 ± 1.27	< 0.001	3.65 ± 1.14	3.62 ± 1.13	0.318	3.65 ± 1.14	3.83 ± 1.17	< 0.001
Bisphenol A	3.16 ± 0.41	3.21 ± 0.41	< 0.001	3.16 ± 0.41	3.19 ± 0.41	0.029	3.16 ± 0.41	3.24 ± 0.42	< 0.001
Triclosan	4.00 ± 0.89	4.08 ± 0.86	0.002	4.00 ± 0.89	3.96 ± 0.87	0.187	4.00 ± 0.89	3.96 ± 0.85	0.246
Butylparaben	2.24 ± 0.78	2.63 ± 0.88	< 0.001	2.24 ± 0.78	2.25 ± 0.80	0.537	2.24 ± 0.78	2.48 ± 0.86	< 0.001
Ethylparaben	3.27 ± 0.79	3.56 ± 0.85	< 0.001	3.27 ± 0.79	3.29 ± 0.80	0.475	3.27 ± 0.79	3.46 ± 0.83	< 0.001
Methylparaben	4.69 ± 0.77	5.02 ± 0.68	< 0.001	4.69 ± 0.77	4.81 ± 0.77	< 0.001	4.69 ± 0.77	4.96 ± 0.73	< 0.001
Propylparaben	3.75 ± 0.99	4.21 ± 0.91	< 0.001	3.75 ± 0.99	3.86 ± 1.00	0.002	3.75 ± 0.99	4.11 ± 0.95	< 0.001

### Association of PNs and PBs metabolite levels and urinary incontinence

A multivariate logistic regression analysis explored the relationship between PN and PB exposure and urinary incontinence. The crude model exhibited a positive correlation between most PNs and PBs exposures and urinary incontinence prevalence (Table 3). After accounting for confounding factors, BP3 exposure displayed a significant association with SUI (OR 1.07 95% CI 1.02-1.14 *p*=0.045), while BPA exposure correlated with UUI (OR 1.21 95% CI 1.01-1.44 *p*=0.046) and MUI (OR 1.26 95% CI 1.02-1.54 p=0.029). TCS exposure demonstrated a significant negative correlation with MUI incidence (OR 0.87 95% CI 0.79-0.97 *p*=0.009). Parabens exposure indicated no significant association with urinary incontinence. Additionally, among females, even with the inclusion of variables such as vaginal deliveries, hysterectomy, and hormone use history (Table 4), BPA exposure remained correlated with MUI prevalence (OR 1.28 95% CI 1.01-1.63 *p*=0.043).

**Table 3.**
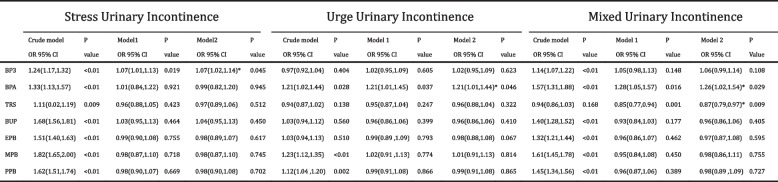
Table 3 Association of urinary incontinence status and parabens&phenols exposure among general population ≥ 20 years of age; NHANES 2007–2016

*OR* Odds ratio, *CI* Confidence intervals. BP3 = Benzophenone-3 BPH = Bisphenol A TRS = Triclosan BUP = Butylparaben EPB = Ethylparaben MPB = Methylparaben PPB = Propylparaben. Crude model: adjusted for none. Model 1: adjusted for age, gender, race, education, BMI. Model 2: adjusted for age, gender, race, education, BMI, cormodity index, alchohol use, smoking status, caffein intake, total water intake, physical activity. *Was marked while *P* < 0.05

**Table 4:** Association of urinary incontinence status and parabens&phenols exposure among female population ≥ 20 years of age; NHANES 2007–2016

	Stress Urine Incontinence		Urge Urine Incontinence		Mixed Urine Incontinence	
	Model3	*P* value	Model3	*P* value	Model3	*P* value
	OR 95% CI		OR 95% CI		OR 95% CI	
Benzophenone-3	1.05(0.98,1.13)	0.170	1.04(0.94,1.15)	0.460	1.03(0.94,1.12)	0.534
Bisphenol A	1.01(0.82,1.26)	0.902	1.25(0.92,1.68)	0.148	1.28(1.01,1.63)	0.043*
Triclosan	1.01(0.91,1.12)	0.841	0.93(0.81,1.07)	0.297	0.91(0.81,1.03)	0.120
Butylparaben	1.02(0.92,1.13)	0.685	0.87(0.75,1.00)	0.058	0.94(0.84,1.06)	0.321
Ethylparaben	1.00(0.90,1.12)	0.947	0.94(0.81,1.08)	0.376	0.98(0.86,1.11)	0.718
Methylparaben	0.96(0.83,1.10)	0.537	0.99(0.82,1.20)	0.882	0.93(0.79,1.08)	0.331
Propylparaben	0.96(0.86,1.06)	0.418	0.97(0.84,1.13)	0.717	0.95(0.84,1.07)	0.374

*OR* Odds ratio

*CI* Confidence intervals

Model 3: adjusted for age, race, education, BMI, cormodity index, alchohol use, smoking status, caffein intake, total water intake, physical activity, vaginal deliveries, hysterectomy, hormone use

^*^was marked while *P* < 0.05

To ascertain any linear trend between urinary incontinence occurrence and parabens and phenol metabolite concentrations, a trend test was conducted. Table 5 illustrates the significant increase in SUI prevalence with rising BP3 exposure levels (OR = 1.21 95% CI = 1.05–1.40 *p* = 0.0082), and a parallel increase in MUI likelihood with elevated BP3 exposure (OR = 1.19 95% CI = 1.02–1.40 *p* = 0.0283).

**Table 5.**
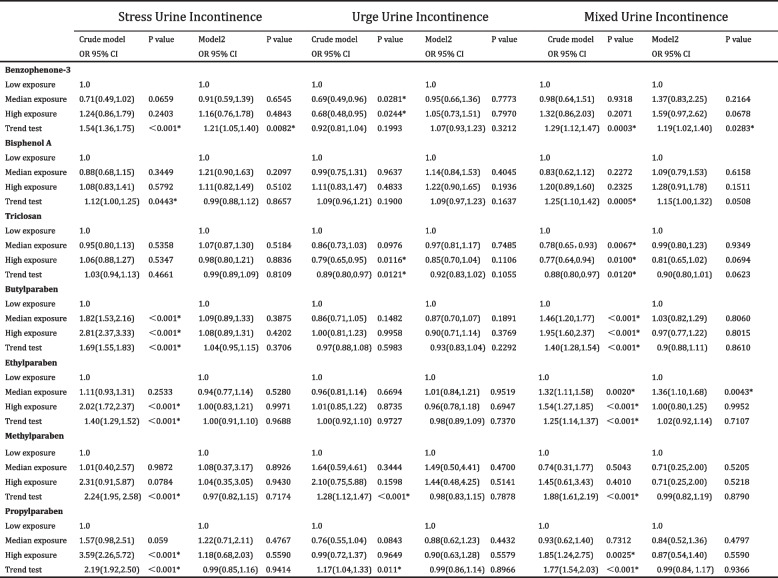
Association of urinary incontinence status and parabens&phenols exposure levels among general population ≥ 20 years of age; NHANES 2007–2016

*OR* Odds ratio. *CI* Confidence intervals. Crude model: adjusted for none. Model 2: adjusted for age, gender, race, education, BMI, cormodity index, alchohol use, smoking status, caffein intake, total water intake, physical activity. *Was marked while *P* < 0.05

### Subgroup analysis on association of PNs and PBs metabolite levels and urinary incontinence

Subgroup analyses (Figure S1-7) unveiled relationships between urinary PNs and PBs and urinary incontinence prevalence stratified by age, BMI, alcohol use, smoking status, and physical activity. Notably, participants aged 65 years and older exhibited a significant association between higher BP3 levels and elevated UUI and MUI prevalence. Further, among normal-weight individuals and specific exposures (BP3, BPA, TCS), distinct correlations with urinary incontinence were observed. Interaction of exposures with alcohol use, smoking status, and physical activity intensity indicated nuanced relationships, shedding light on the complexity of urinary incontinence etiology.

### Association between PNs and PBs mixture and urinary incontinence by the BKMR model

**Fig. 2:**
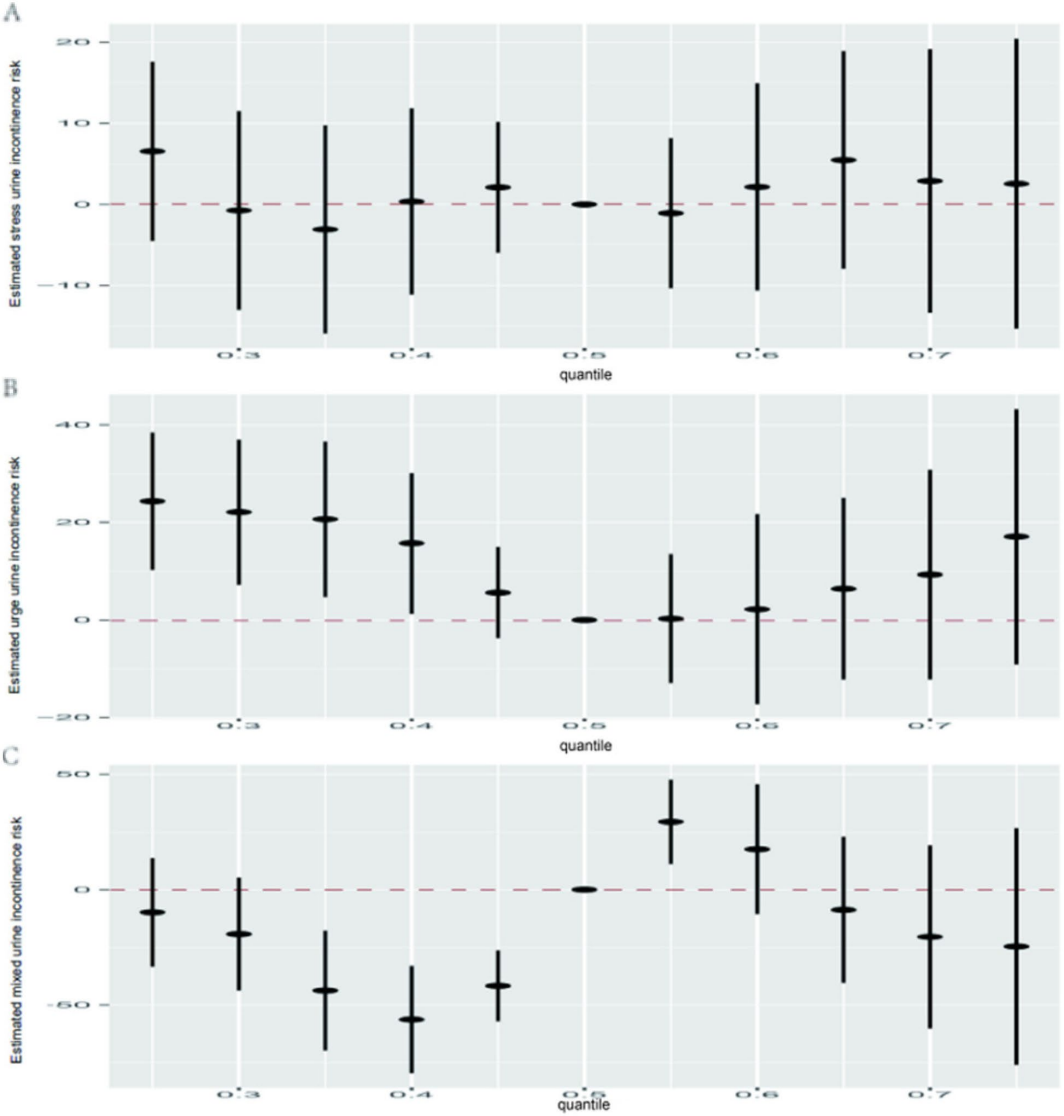
Joint effects of urine phenols and parabens mixture on SUI (**A**), UUI(**B**), and MUI(**C**) risk in the general population. Models were adjusted for age, gender, race, education, BMI, comorbidity index, alcohol use, smoking status, caffeine intake, total water intake, and physical activity. The Y-axis represents the estimated difference in z-scores when all metabolites were fixed at specific quantiles (ranging from 0.25 to 0.75), as compared to when metabolites were at the 50th percentile. Dots indicate the estimate, and black vertical lines represent 95% CIs

In the context of SUI, the 95% CI for overall effect estimates in the SUI model all encompassed 0, suggesting no significant association (Fig. 2A). Regarding UUI, a noteworthy negative trend was identified in the risk of UUI with the concentration of the mixture. This trend was particularly significant when all PN and PB metabolites were in the range of their 25th to 40th percentiles, as opposed to when they were all at their 50th percentile (Fig. 2B), in which BPA (cond PIP = 0.668) played the most important role (Table S1). Concerning MUI, our analysis revealed a favorable inclination between the mixture of PN and PB metabolites and the risk of UUI. This tendency was prominent when the mixture ranged from the 40th to the 55th percentile, in contrast to when it was at the 50th percentile. Additionally, an opposing tendency was observed between the mixture of PN and PB metabolites and UUI when the mixture ranged from the 35th to the 40th percentile (Fig. 2C), TCS, and BPA were selected for inclusion in more than 50% of iterations when we added variable selection in the BKMR models (Table S1).

## Discussion

We present evidence for the significantly higher concentration of BP3 among patients with SUI. Aoki et al.'s work [3] underscores the pivotal role of urethral hypermobility and urinary sphincter weakness in the pathogenesis of SUI, attributing urethral hyperactivity to the loss of support caused by weakened endopelvic fascia and muscles during episodes of abdominal pressure. Notably, sex steroid hormones exert a profound influence over pelvic muscle and connective tissue synthesis and metabolism [19], suggesting that BP3's potential to disrupt pelvic floor muscle function might result from the modulation of hormone dynamics. However, postmenopausal women participating in human studies do not show significant associations between urinary BP-3 concentration and reproductive hormone levels, including estradiol, progesterone, follicle-stimulating hormone, and luteinizing hormone [20]. Furthermore, reports indicate decreased plasma testosterone levels after applying BP-3-containing creams to both adolescents and adults [21]. Mammadov et al.'s findings [22] corroborate the notion of pelvic floor muscle atrophy improvement following testosterone administration in SUI models, suggesting testosterone's anabolic effect on pelvic floor muscles. This aligns with Bhasin et al.'s observations [23] of androgens enhancing the strength of almost all striated muscles, including the levator ani muscle crucial for pelvic floor support. As both endogenous and exogenous androgens bolster pelvic floor function, BP3's disruption of testosterone function could potentially increase SUI risk. Additionally, neurogenic damage may underlie urinary sphincter functional decline. Wnuk et al.'s work [24] also highlights BP3's potential to alter receptor expression and impede necessary nervous system functions, thus implicating the long-term accumulation of BP-3 in SUI pathogenesis through neural interference. Collectively, BP3's interplay with hormonal dynamics and neurologic integrity could contribute to SUI etiology.

Research demonstrates a positive relationship between urinary BPA levels and increased probabilities of both UUI and MUI. Notably, the association between BPA exposure and increased MUI risk persists in the female demographic. UUI, characterized by detrusor muscle overactivity, leads to involuntary urine leakage. Mice exposed to BPA exhibit voiding dysfunction, marked by urethral histological changes and exacerbated non-voiding contractions due to detrusor instability [16]. This offers evidence of BPA's potential to impair detrusor function and contribute to UUI development. Doumouchtsis et al.'s review [25] underscores the intimate connection between obesity and urinary incontinence, advocating weight loss for its mitigation. A comprehensive meta-analysis across ten studies reveals a dose–response relationship between BPA exposure and the risk of obesity, indicating that a 1 ng/mL increase in BPA correlates with an 11% rise in obesity risk [26]. This implies that BPA may contribute to obesity, eventually leading to urinary incontinence in the general population. Moreover, chronic abdominal pressure, a hallmark of obesity, could compromise the pelvic floor. Furthermore, adipose tissues releasing oxidative stress factors may contribute to the degradation of pelvic floor structures [25]. Notably, MUI is diagnosed based on the co-occurrence of UUI and SUI. Although our study reveals BPA's association with UUI and MUI, the lack of association with SUI suggests a complex interplay underlying MUI, beyond a mere summation of UUI and SUI mechanisms. This emphasizes the need for further exploration to unveil the intricate determinants of MUI development.

Interestingly, heightened TCS exposure seems inversely linked to MUI occurrence in the general population. First, TCS exposure closely correlates with increased production of reactive oxygen species (ROS) in humans [27]. Prolonged ROS accumulation is linked to tissue and organ inflammation, indicating a potential avenue. Aoki et al.'s review [3] emphasizes persistent urothelial irritation from oxidative stress as a central mediator of bladder overactivity, supporting the role of ROS. Second, earlier studies [28] describe a simultaneous reduction in BMI with increased urinary TCS concentrations. As obesity has the potential to compromise pelvic floor muscles and diminish pelvic viscera support [25], TCS's impact on BMI might contribute to its effect on MUI. Meanwhile, incontinent postmenopausal women exhibit significantly lower serum Δ4-androstenedione levels and androgen receptors, which are commonly verified by biopsies from the bladder neck, and urethra compared to control subjects [29, 30]. In vitro experimentation by Christen et al. [31] illuminates augmenting dihydrotestosterone response within limited concentrations in MDA-kb2 cells co-treated with dihydrotestosterone and TCS. This suggests that TCS's dose-dependent androgen receptor agonism may contribute to enhancing androgen activity in pelvic floor muscles, potentially protecting against urinary incontinence. Additionally, TCS's impact on calcium signaling, evident in increased cytosolic calcium levels in resting myotubes [32], suggests potential roles in regulating muscle function. In aggregate, these factors collectively propose TCS's multifaceted engagement in MUI modulation.

Our study did not find a clear association between paraben exposure and any form of UI. Similar to other Endocrine Disrupting Compounds (EDCs), parabens interfere with sex hormone function and aromatase activity [33, 34]. In a cohort study [33], a correlation is established between elevated paraben levels and reduced estradiol levels. Nevertheless, Nowak et al. [35] highlight inconsistencies between animal models and human studies regarding paraben effects on the endocrine system. This is attributed to exceptionally high experimental doses in animals, leading to unreliable extrapolations of human outcomes. Kim et al.'s findings [36] suggest a significant concentration disparity between animal and in vitro studies, and actual human tissue measurements. Moreover, analysis of the Canadian Health Measures Survey [36] reveals a gender-dependent association between parabens and obesity, particularly in females, hinting at metabolic and sex hormone disparities in paraben exposure outcomes. In summary, it seems that paraben concentrations in humans may be insufficient to cause UI, despite their potential to disrupt the endocrine system.

BKMR models pinpointed urinary BPA, BP3, and TCS as the primary contributors to the overall effect. However, the impact on UI seems to vary at different concentrations, showing a slight deviation from the results of the regression analysis. This implies that comprehending the counteracting effects among exposures is crucial when evaluating the impact of mixtures on UI. This underscores that examining only one factor in isolation is insufficient for understanding the fundamental pathogenesis. It emphasizes the necessity for additional basic research to explore the combined effects of common substances on the same signaling pathway [37].

Our investigation reveals the potential involvement of BPA, TCS, and BP3 in the pathogenesis of urinary incontinence through mechanisms such as hormonal imbalance, inflammation, oxidative stress, and obesity. However, due to concentration disparities between in vitro and human studies, parabens' association with UI appears limited. These findings highlight the need for comprehensive research to enhance diagnostic precision, treatment strategies, and quality of life for urinary incontinence patients. Further investigations are warranted to provide nuanced insights into the complex landscape of UI etiology and progression.

Naturally, there are also some limitations. Our cross-sectional study doesn't establish causation; the observed associations may not imply a cause-and-effect relationship. Reverse causality, where outcomes influence exposures, is possible. Relying on self-reported patient data for diagnosing urinary incontinence may introduce misdiagnosis and recall bias. Unaccounted factors like pelvic surgery and urinary tract infections in the multifaceted nature of urinary incontinence could potentially influence our adjusted model. Limited information on personal care product usage frequency hinders exploring associations with urinary incontinence outcomes. Lastly, the rapid metabolism of PNs and PBs in the body poses a limitation, as our single sampling instance may not fully capture their long-term effects, which can vary over time.

## Conclusion

In summary, our study revealed significant associations between UI and certain compounds in US adults. Specifically, we found positive associations with urinary BPA and BP-3 levels, while urinary TCS showed an inverse correlation. However, urinary parabens did not show significant associations with UI. The observational nature of our study necessitates caution in drawing causal conclusions. Future research using prospective study designs is crucial to validate our findings and deepen our understanding of the mechanisms linking PNs and PBs to UI, providing a more comprehensive insight into these complex associations.

### Supplementary Information

**Additional file 1. **

## Data Availability

The raw data supporting the conclusions of this article can be found here: https://www.cdc.gov/nchs/nhanes/.

## Abbreviations

NHANES: National Health and Nutritional Examination Survey
PN: Phenol
PB: Paraben
TCS: Triclosan
BPA: Bisphenol A
BP-3: Benzophenone-3
BPB: Butylparaben
EPB: Ethylparaben
MPB: Methylparaben
PPB: Propylparaben
SUI: Stress urinary incontinence
UUI: Urge urinary incontinence
MUI: Mixed urinary incontinence
BKMR: Bayesian kernel machine regression
