# Supplementary material for: Urinary phenols and parabens exposure in relation to urinary incontinence in the US population
Source: BMC Public Health. 2024 Feb 19;24:515. doi: 10.1186/s12889-024-17872-9 (PMC10875867; doi:10.1186/s12889-024-17872-9)
Supplement: Supplementary file 1 — Additional file 1. [file 12889_2024_17872_MOESM1_ESM.docx]

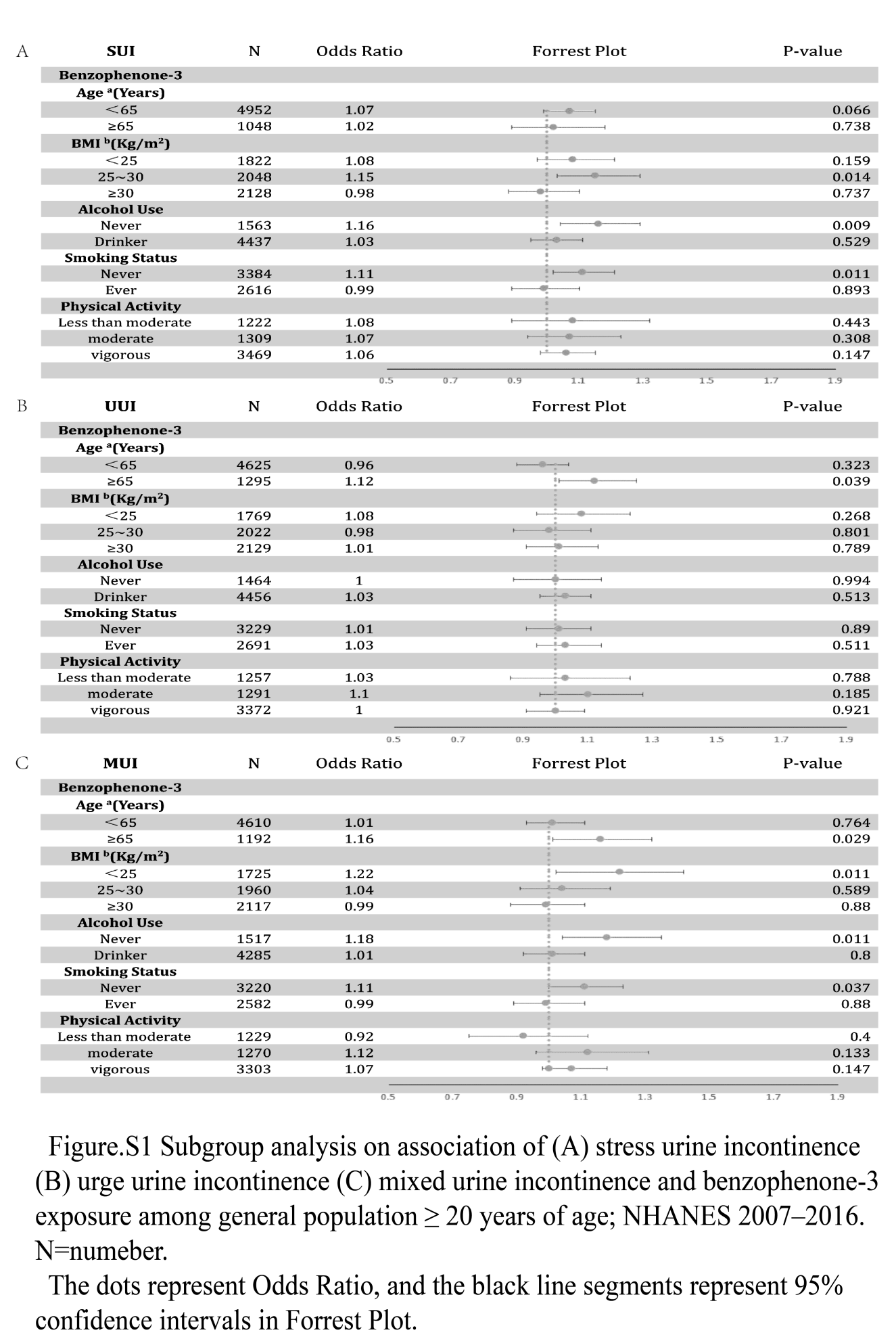


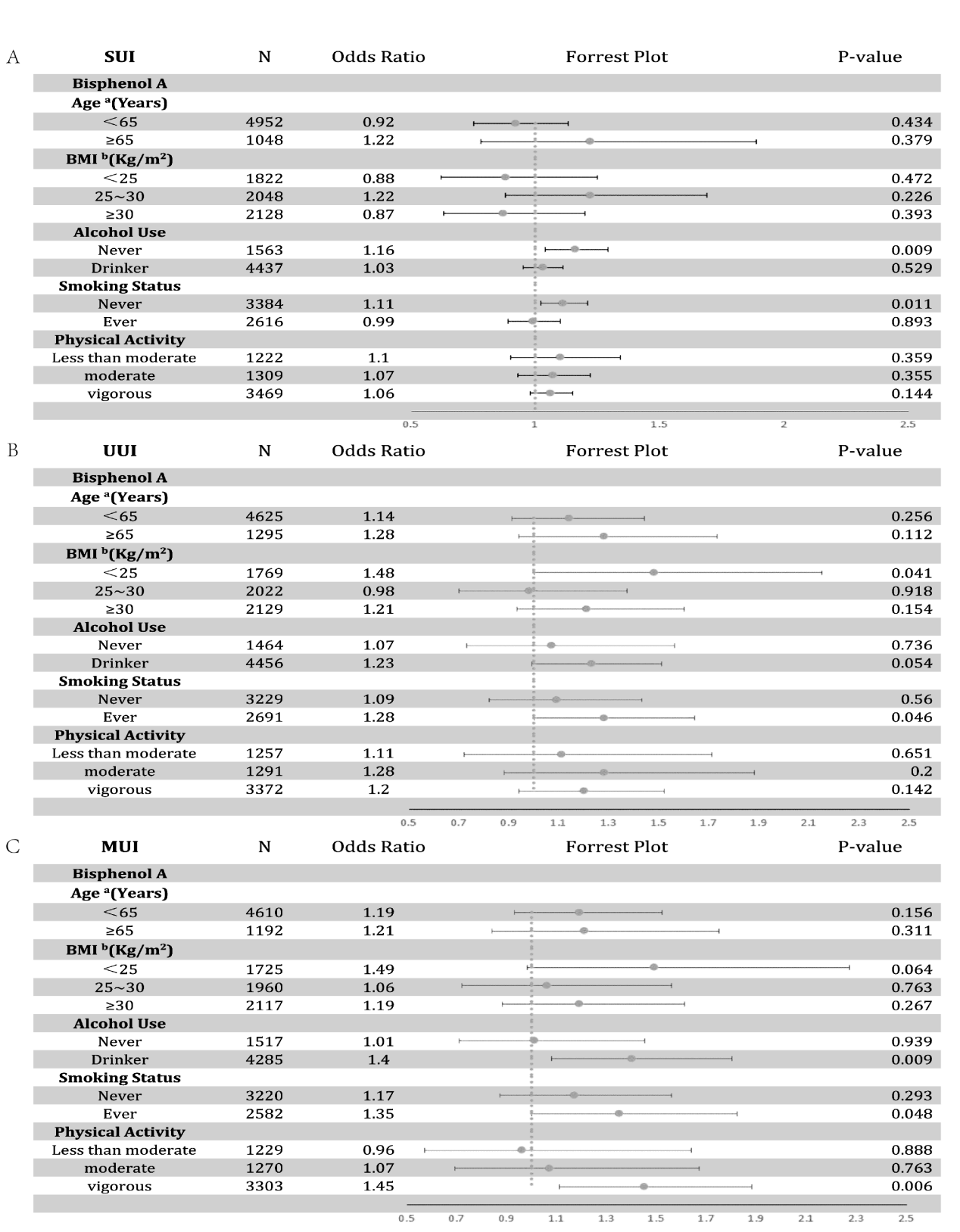


Figure.S2 Subgroup analysis on association of (A) stress urine incontinence (B) urge urine incontinence (C) mixed urine incontinence and bisphenol A exposure among general population ≥ 20 years of age; NHANES 2007–2016.

N=number

The dots represent Odds Ratio, and the black line segments represent 95% confidence intervals in Forrest Plot.


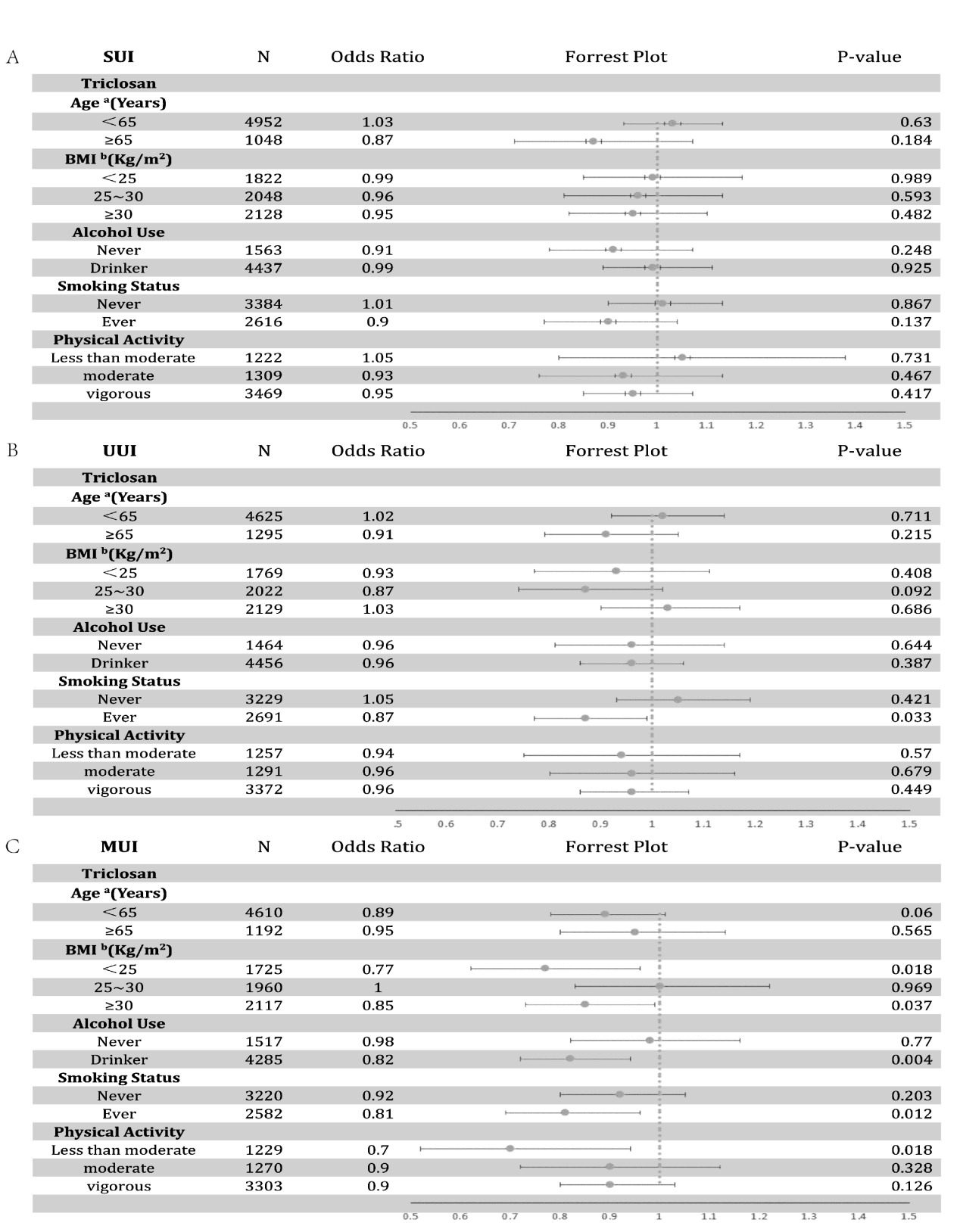


Figure.S3 Subgroup analysis on association of (A) stress urine incontinence (B) urge urine incontinence (C) mixed urine incontinence and Triclosan exposure among general population ≥ 20 years of age; NHANES 2007–2016.

N=number

The dots represent Odds Ratio, and the black line segments represent 95% confidence intervals in Forrest Plot.


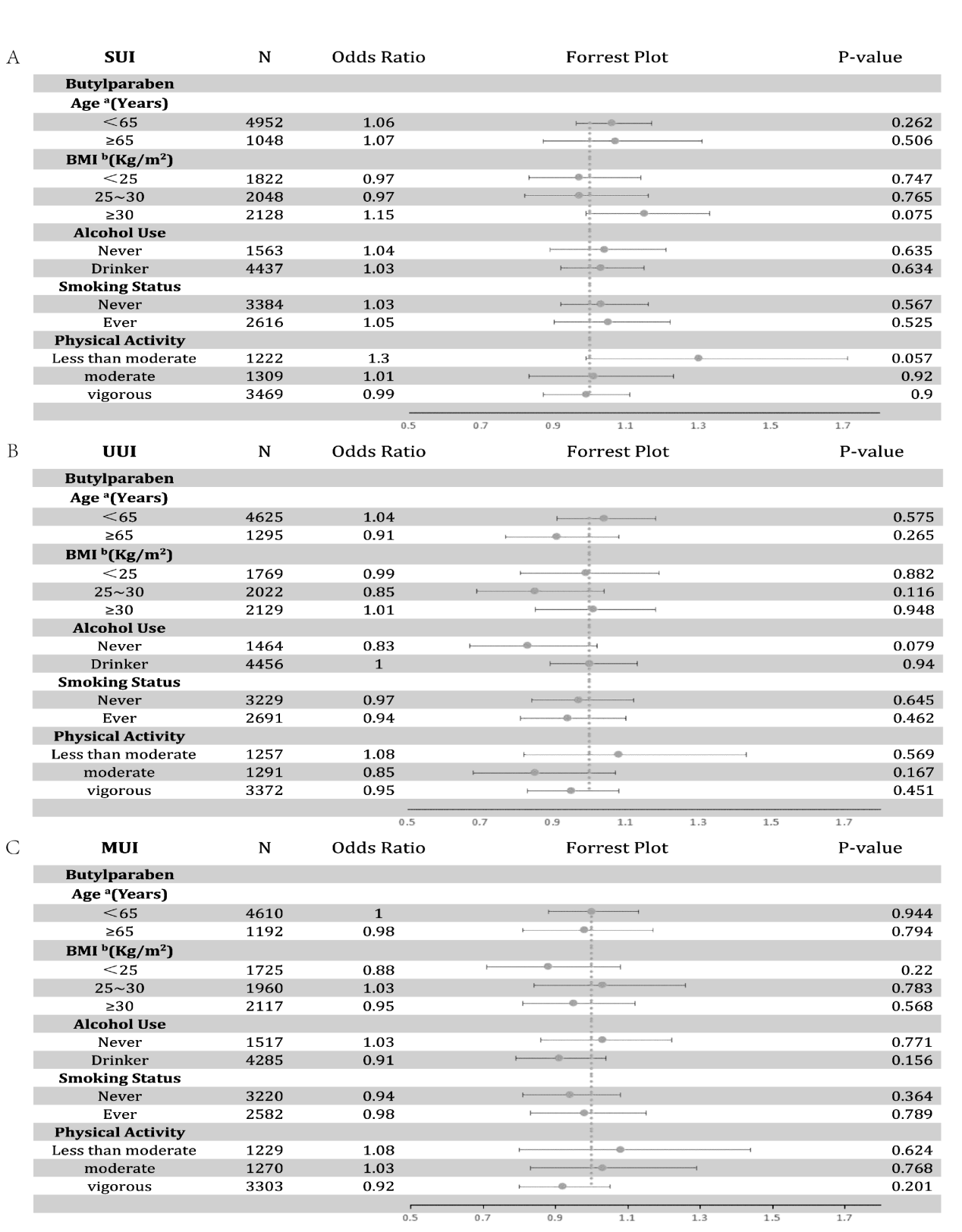


Figure.S4 Subgroup analysis on association of (A) stress urine incontinence (B) urge urine incontinence (C) mixed urine incontinence and butylparaben exposure among general population ≥ 20 years of age; NHANES 2007–2016.

N=number

The dots represent Odds Ratio, and the black line segments represent 95% confidence intervals in Forrest Plot.


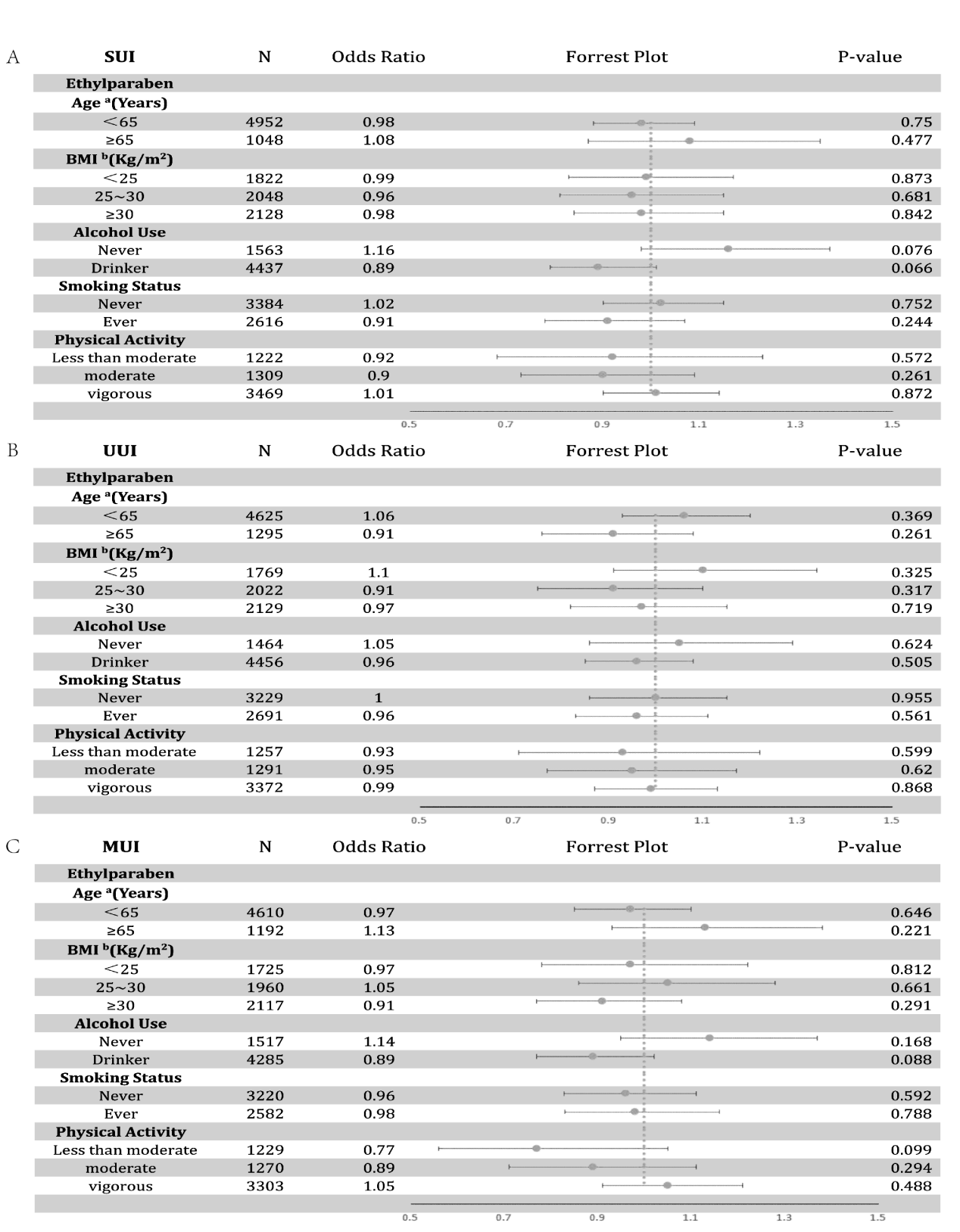


Figure.S5 Subgroup analysis on association of (A) stress urine incontinence (B) urge urine incontinence (C) mixed urine incontinence and ethylparaben exposure among general population ≥ 20 years of age; NHANES 2007–2016.

N=number

The dots represent Odds Ratio, and the black line segments represent 95% confidence intervals in Forrest Plot.


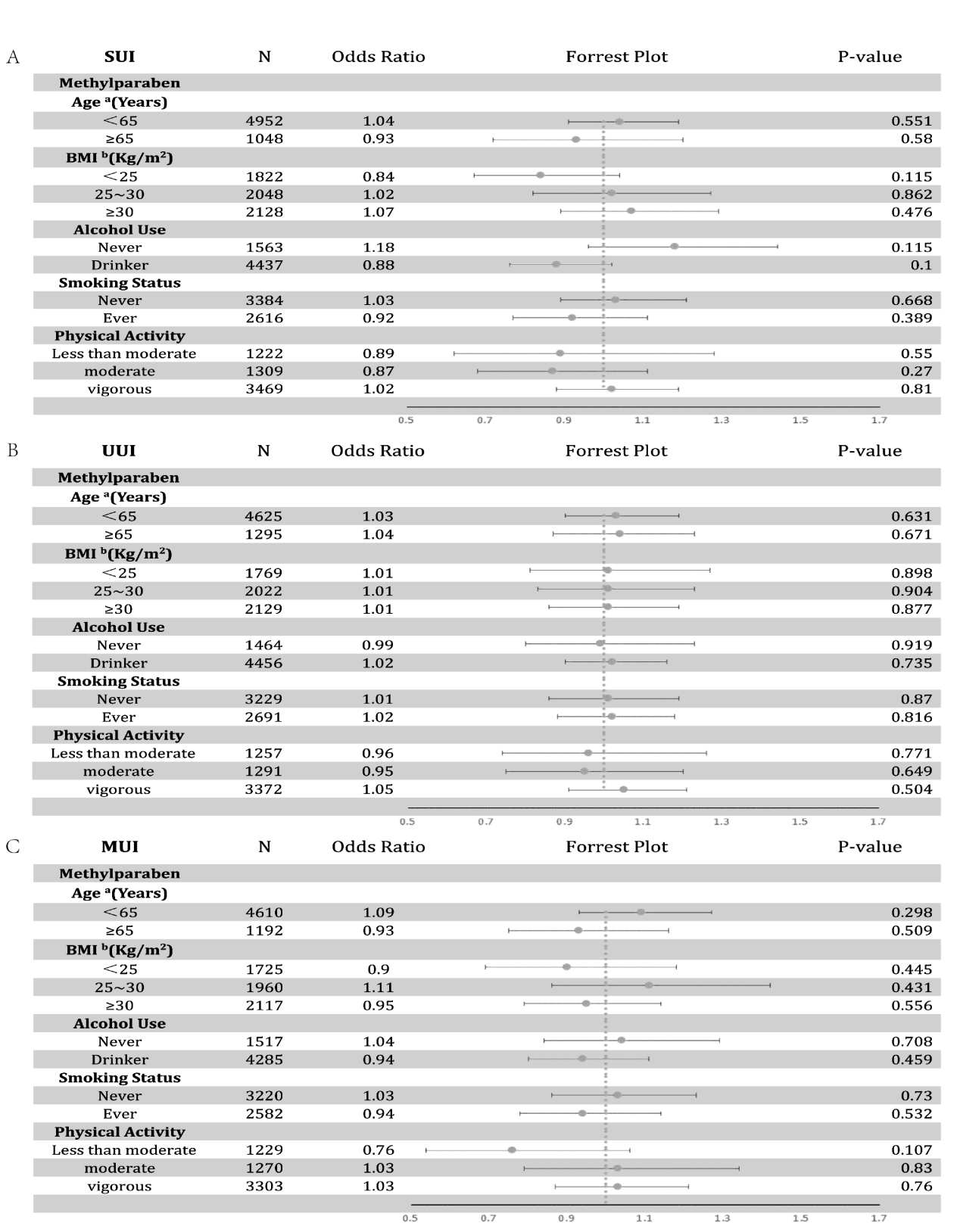


Figure.S6 Subgroup analysis on association of (A) stress urine incontinence (B) urge urine incontinence (C) mixed urine incontinence and methylparaben exposure among general population ≥ 20 years of age; NHANES 2007–2016.

N=number

The dots represent Odds Ratio, and the black line segments represent 95% confidence intervals in Forrest Plot.


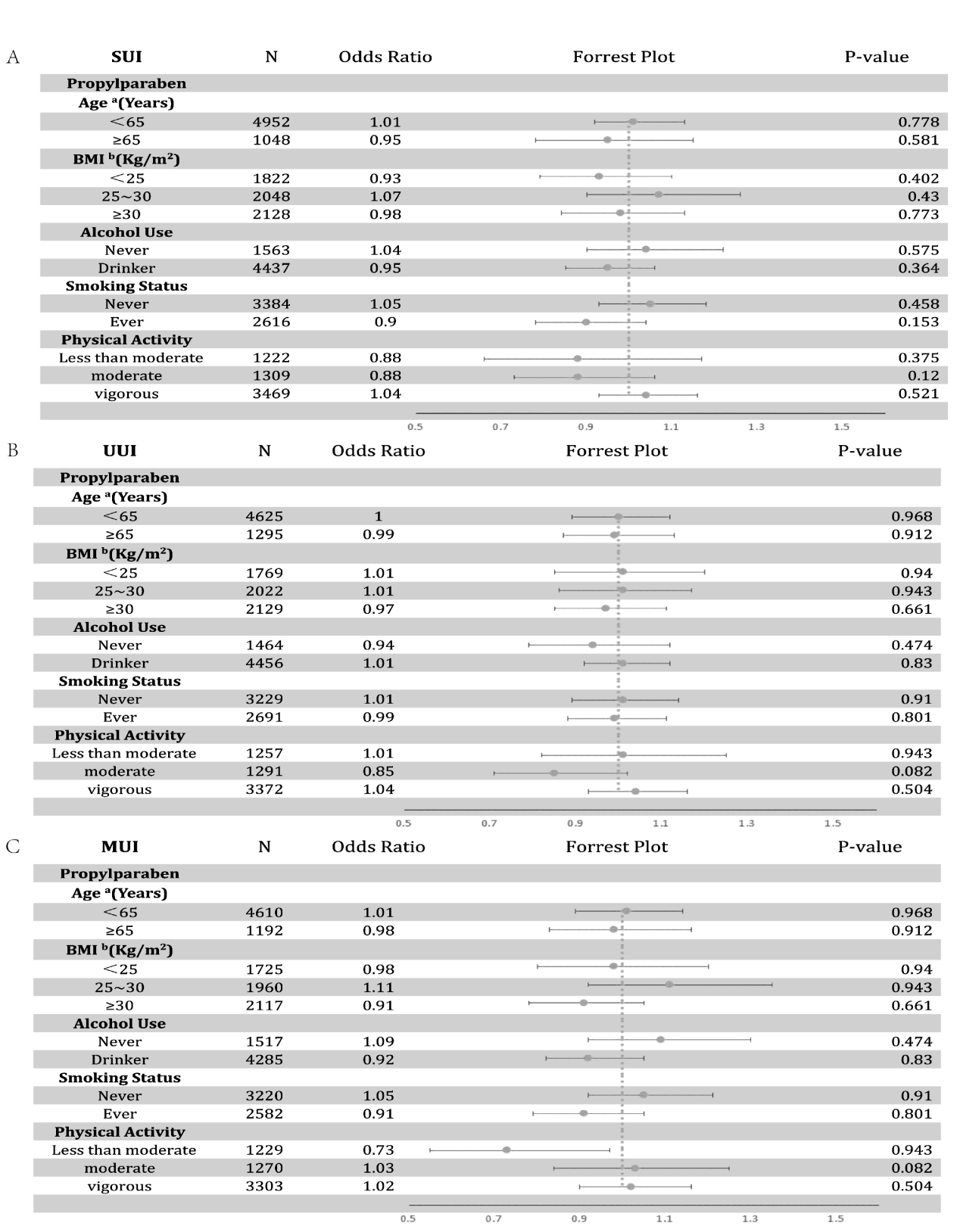


Figure.S7 Subgroup analysis on association of (A) stress urine incontinence (B) urge urine incontinence (C) mixed urine incontinence and propylparaben exposure among general population ≥ 20 years of age; NHANES 2007–2016.

N=number

The dots represent Odds Ratio, and the black line segments represent 95% confidence intervals in Forrest Plot.

Table.S1 Posterior inclusion probabilities (PIPs) derived from BKMR model for conditional inclusion into urine incontinence models.

|  | Stress Urine Incontinence |  | Urge Urine Incontinence |  | Mixed Urine Incontinence |  |
| --- | --- | --- | --- | --- | --- | --- |
|  | conPIP | | conPIP | | conPIP | |
| Benzophenone-3 | 0.752  0.265  0.501  0.127  0.223  0.224  0.368 | | 0.423  0.668  0.501  0.106  0.223  0.361  0.368 | | 0.423  0.841  0.723  0.168  0.295  0.236  0.412 | |
| Bisphenol A |  |  |  |  |  |  |
| Triclosan |  |  |  |  |  |  |
| Butylparaben |  |  |  |  |  |  |
| Ethylparaben |  |  |  |  |  |  |
| Methylparaben |  |  |  |  |  |  |
| Propylparaben |  |  |  |  |  |  |

Note : adjusted for age, race, education, BMI, cormodity index, alchohol use, smoking status, caffein intake, total water intake, physical activity, vaginal deliveries, hysterectomy, hormone use.
